# Edible Seaweeds and Spirulina Extracts for Food Application: In Vitro and In Situ Evaluation of Antimicrobial Activity towards Foodborne Pathogenic Bacteria

**DOI:** 10.3390/foods9101442

**Published:** 2020-10-12

**Authors:** Francesco Martelli, Martina Cirlini, Camilla Lazzi, Erasmo Neviani, Valentina Bernini

**Affiliations:** Department of Food and Drug, University of Parma, Parco Area delle Scienze 49/A, 43124 Parma, Italy; francesco.martelli@studenti.unipr.it (F.M.); martina.cirlini@unipr.it (M.C.); camilla.lazzi@unipr.it (C.L.); erasmo.neviani@unipr.it (E.N.)

**Keywords:** food safety, natural antimicrobial, algae extract, phenolic content, *Listeria monocytogenes*, foodborne pathogenic bacteria, microbiological challenge test

## Abstract

Research is more and more focused on studying and selecting food preservatives of natural origin. In this scenario, algae are an excellent source of bioactive compounds, among which are antimicrobials, whose presence is variable depending on the algal species and environmental conditions. The aim of the present study was to obtain, by a food grade approach, antimicrobial extracts from five species already approved as foods and to test their efficacy in vitro (agar well diffusion assay) and in situ (microbial challenge test) towards foodborne pathogenic bacteria. Moreover, the total phenolic compounds of the extracts were determined in order to evaluate possible correlations with the antimicrobial activity. Strains belonging to *Salmonella* spp., *Listeria monocytogenes*, *Escherichia coli*, *Staphylococcus aureus*, and *Bacillus cereus* were considered. Overall, the extracts showed a good antimicrobial activity in vitro towards all the tested microorganisms, especially *L. monocytogenes* (15 mm of inhibition diameter). The effect of inhibition was monitored during 24, 48 and 120 h showing a good persistence in time. *Arthrospira platensis* exerted the highest efficacy, further revealed towards *L. monocytogenes* on salmon tartare as bacteriostatic using 0.45% of the extract and bactericidal using 0.90%. The presence of phenolic compounds could be related to the antimicrobial activity but was not revealed as the main component of this activity. The extract with the highest phenolic content (18.79 ± 1.90 mg GAE/g) was obtained from *Himanthalia elongata*. The efficacy, confirmed also in a food matrix, might open perspectives for their application as food preservative.

## 1. Introduction

Seaweeds and microalgae are becoming progressively popular as food in western countries, and some species in particular are recognized as a valuable source of healthy compounds [1,2,3]. Algae are indeed a good supply of bio-actives and functional constituents for diet, such as essential amino acids, polyunsaturated fatty acids, fibers, vitamins, phytosterols and minerals [4]. Twenty million tons of seaweeds are harvested worldwide every year and half of the production is destined for human consumption in the form of dried products [5]. *Arthrospira platensis*, an edible cyanobacterium commercially known as spirulina, is also commonly employed as food supplement because of its nutritional value [6,7]. Many bioactivities have been attributed to algae including antioxidant, antidiabetic, anti-inflammatory and immunomodulation actions. Lately, many studies involving the implementation of algae in food formulations to obtain or enhance functional and nutritional properties of food have been presented [8,9,10].

Antimicrobial activity, among other properties, has been proved for some algae [11,12,13,14,15] even if to a variable extent according to several factors such as: (i) species; *Phaeophyceae* for instance are described as the most effective against foodborne pathogenic bacteria; (ii) chemical composition, which varies according to collecting area and season; (iii) type of solvent used in the extraction process; indeed, ethanolic and methanolic extracts are very effective against Gram-negative and Gram-positive bacteria; (iv) concentration of the extract [16]. Some algal species and bioactive molecules extracted fall into the category of “novel food” introduced by Regulation (EC) 258/97. This applies to all food products and substances that were not significantly consumed until 15 May 1997 within the European Union. This Regulation was subsequently replaced by the new Regulation (EU) 2015/2283, which simplifies the conditions for introducing new foods in the EU market while maintaining a high level of food safety [17]. The algal species used in this work are already approved as food by EFSA (European Food Safety Authority), making easier the use of their extract in food formulations.

The proliferation of foodborne bacterial pathogens endanger consumers’ safety since these microorganisms are responsible for infections or intoxications. Preservatives are commonly used in food products to inhibit microbial growth but consumers’ concern due to their possible harmful effects on health has generated an increasing demand for foods free from synthetic additives [18]. Therefore, the role of algal extracts as antimicrobial agents against foodborne pathogens might guarantee food safety fulfilling the requests of consumers for products with a “clean labels” status.

In this context the aim of the present study was to: (i) obtain antimicrobial extracts from algae approved as food, such as *Himanthalia elongata, Laminaria* spp., *Palmaria palmata* and *Undaria pinnatifida*, using food grade extraction, and test their efficacy in vitro towards the main foodborne pathogens (*Salmonella* spp., *L. monocytogenes*, *E. coli*, *S. aureus*, and *B. cereus*), by agar well diffusion assay; (ii) evaluate in situ, on salmon tartare as food model, the efficacy of the best performing extract by microbiological challenge test; (iii) evaluate the role of total phenolic compounds of the extracts on antimicrobial activity.

## 2. Materials and Methods

### 2.1. Algae Samples Preparation

Four species of seaweed (*H. elongata, Laminaria* spp., *P. palmata* and *U. pinnatifida*) and a cyanobacterium (*A. platensis)* were considered. Dried samples were purchased on the Italian market considering two different producers for each species, in order to evaluate variability. The ten samples were grinded with Oster 890-48H mixer (Recampro, Rianxo, Spain) and maintained at room temperature in darkness until use.

### 2.2. Extraction Process

With the purpose of extracting compounds with potential antimicrobial activity such as small peptides, polyphenols and organic acids, an extraction process was carried out as described by Martelli et al. (2020) [19]. In particular, 10 g of grinded sample were extracted with 100 mL of ethanol/water (70:30 *v*/*v*) acidified with 1% formic acid (CH_2_O_2_). A double extraction was performed, twice, alternating a shaking cycle in an HS 501 digital shaker (IKA) (IKA-Werke GmbH & Co, Staufen, Germany) at 200 strokes/minute with a sonication cycle in an Ultrasonic Cleaner sonicator (VWR, Radnor, PA, USA), each lasting 15 min. The sample was then centrifuged (Eppendorf 5800 Centrifuge, Model 5810R, Hamburg, Germany) at 12,857× *g* for 10 min at 10 °C. The solution was filtered on a paper filter to recover the solid part so as to proceed to the second extraction. The two extracts obtained were combined and dried under vacuum on a Strike 300 rotary evaporator (Steroglass, Perugia, Italy) at 4× *g* with a bath temperature of 40 °C. The dried extracts obtained were dissolved in sterile bi-distilled water to obtain 250 mg/mL solutions. For each species, two extracts were obtained (I and II). All the extractions were carried out in duplicate in order to test the reliability of the extraction method.

### 2.3. Foodborne Pathogenic Strains

The antimicrobial activity of the extracts was tested towards 14 strains belonging to the main foodborne pathogenic bacteria: *Salmonella* spp. (*S. enterica* ATCC 14028; *S. enterica* serotype *Rissen* and *Salmonella* spp. suini), *Listeria monocytogenes* (LM30; LMG 21,264 and LMG 13305), *Escherichia coli* (DSM 9025; DSM 10,973 and POM 1048), *Staphylococcus aureus* (NCTC 9393; ATCC 6538 and ATCC 19095) and *Bacillus cereus* (31 and 33). These strains belong to the collection of the Department of Food and Drugs (University of Parma, Parma, Italy) and to international collections: National Collection of Type Cultures (NCTC), Belgian Co-ordinated Collection of Microorganisms (BCCM), American Type Culture Collection (ATCC), and Deustsche Sammlung von Mikroorganismen (DSM). Bacteria were kept at −80 °C in Tryptone Soya Broth (TSB) (Oxoid, Basingstoke, UK) supplemented with 12.5% glycerol (*v*/*v*). Before use, they were revitalized twice by inoculum (3% *v*/*v*) in TSB (Oxoid) supplemented with 0.6% of yeast extract and then incubated for 16 h at 37 °C in aerobic conditions.

### 2.4. Evaluation of In Vitro Antimicrobial Activity

An agar well diffusion assay was carried out in order to evaluate the antimicrobial activity of the extracts [19,20]. The pathogenic bacteria were diluted to a concentration of 8 Log CFU/mL and seeded on Tryptone Soya Agar (TSA) (Oxoid) by using sterile swabs. Then, wells having a diameter of 7 mm were created by means of sterile tips in the agar and filled with 30 μL of each extract. The antimicrobial activity was evaluated by measuring the diameter of the inhibition zone (mm) after 24, 48 and 120 h of incubation at 37 °C in aerobic conditions. Water was used as negative control. The analyses were performed in triplicate and average values ± standard deviations were reported.

### 2.5. Microbiological Challenge Test

Specific studies are recommended to gather experimental data in case the behaviour of a microorganism in a product is not well known [21]. For this purpose, a microbiological challenge test (MCT) can evaluate if an artificially inoculated organism can survive or develop in a specific product, determining the eventual arriving at unacceptable levels [22]. In the case of *L. monocytogenes* the refrigerated storage cannot be considered a sufficient parameter to inhibit its growth [23] and MCT becomes an important tool to monitor its behavior in ready-to-eat (RTE) foods and to evaluate the efficacy process parameters or ingredients used as preservatives. In the present work the extract that has shown the highest in vitro efficacy was selected to test the antimicrobial activity in situ. The test was conducted as described by Bernini et al. (2013) with some modifications [24]. A MCT was designed in salmon tartare, chosen as a food model, considering two different extract concentrations, 0.45 and 0.9% (*v*/*w*), corresponding to 4.5 mg/g and 9.0 mg/g respectively in the product. Negative samples, consisting of salmon tartare without any addition of the extract, were also considered. The antimicrobial activity was tested towards a mixture of the three *L. monocytogenes* strains used for the in vitro test (LM30, LMG 21264 and LMG 13305) and revitalized as previously described (2.3). The salmon tartare, with the extract added or not, was then contaminated in order to obtain a final concentration of 4 Log CFU/g of *L. monocytogenes* in the product. The tartare was then mixed to distribute evenly the contamination, portioned, and stored at refrigeration temperatures for four days to simulate a domestic conservation. *L. monocytogenes* was monitored just after inoculum (T0), after 24 h (T1), 48 h (T2), 72 h (T3) and 96 h (T4) of shelf life by plate count on Agar Listeria acc. Ottaviani & Agosti (ALOA) (Biolife, Milan, Italy) after incubation at 37 °C for 24 h. Analyses were performed in triplicate and average values ± standard deviations were reported.

### 2.6. Chemicals

Ethanol, methanol and formic acid used for extraction procedures were obtained from Sigma-Aldrich (St. Louis, MO, USA), while bi-distilled water was in-house produced by a Millipore Alpha Q purification system (Waters, Billerica, MA, USA). Sodium carbonate and gallic acid utilized for the determination of phenol content were purchased from Sigma-Aldrich (St. Louis, MO, USA), while Folin-Ciocalteu’s phenol reagent solution was obtained from VWR (Milano, Italy).

### 2.7. Total Phenolic Amount Determination

The determination of the total phenolic content was performed following the protocols reported by Cox et al. (2010) [25] with some modifications. In particular, 0.10 g of seaweed extract were weighted, added with 10 mL of a methanol/bi-distilled water solution (70/30, *v*/*v*) and extracted at room temperature by constant shaking on a shaker HS 501 digital (IKA, Staufen, Germany) at 200 strokes/minute for 30 min. Then, the solutions were centrifuged at 5000 rpm for 10 min at room temperature using a Centrifugette 4206 centrifuge (Alc International, Pévy, France). 200 µL of sample extracted were then added to 1 mL of Folin-Ciocalteu’s phenol reagent solution, previously diluted in bi-distilled water (1/10, *v*/*v*), and 2 mL of aqueous sodium carbonate (20%, *w*/*v*), and incubated in the dark for 30 min. After this step, the absorbance of the solution was measured at 760 nm by a JASCO V-530 spectrophotometer (Easton, MD, USA). Blank was prepared and analysed following the same procedure. The total phenolic amount was calculated as mg gallic acid equivalent per g (mg GAE/g) from a calibration curve obtained by measuring the absorbance at 760 nm of 5 gallic acid solutions at different concentrations: 0.01, 0.025, 0.05, 0.075 and 0.1 mg/g. All the extractions and analyses were repeated twice, while for each extract and standard solution the absorbance measurement was performed in triplicate.

### 2.8. Statistical Analysis

The statistical analysis was carried out by testing a univariate multifactorial ANOVA using the GLM software SAS University edition (SAS, Cary, NC, USA). With the significance test (*p* value < 0.05) a first analysis was carried out in which the inhibition halo was placed as a dependent variable, while extract, contact time and algae species corresponded to the independent variables. Subsequently, a post-hoc TEST (Tukey) was carried out. The antimicrobial activity of the different extracts obtained from the same algae species (I and II) was compared and evaluated against each pathogenic strain at different contact times. Statistical elaborations of the results obtained from total phenolic content determination were performed with IBM SPSS Statistics 23 (IBM, New York, NY, USA). In particular, two tailed *t*-test for independent samples was applied to determine a significant difference (*p* < 0.05) between extracts I and II corresponding to different samples of the same algae species. Conversely, to define analogies and/or differences among samples belonging to different species, one-way ANOVA analysis was applied using Tukey test and the results were considered different for values of *p* < 0.05.

## 3. Results

### 3.1. In Vitro Antimicrobial Activity towards Foodborne Pathogens

**Figure 1:**
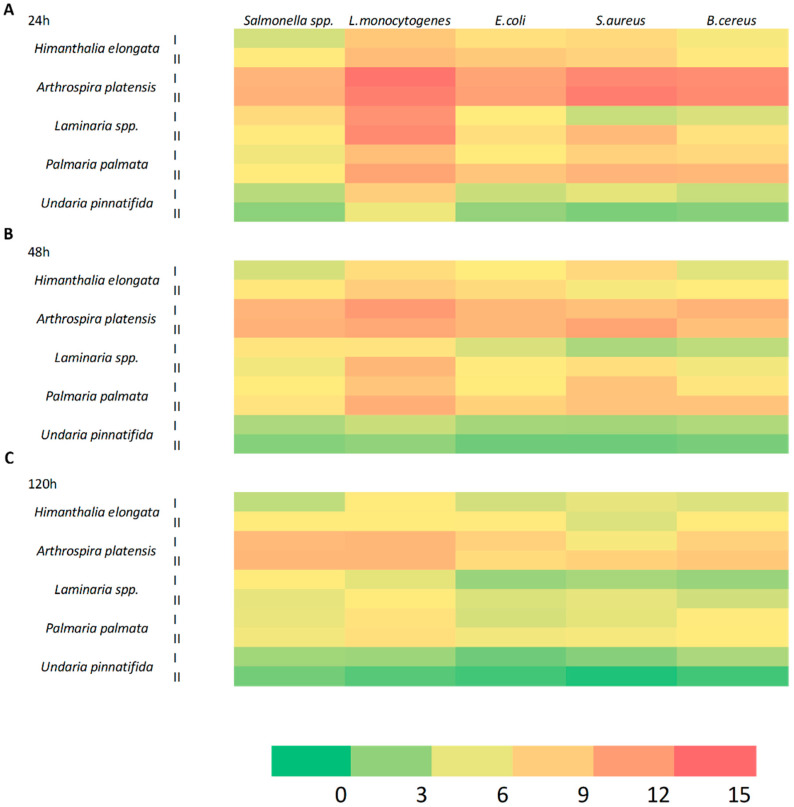
Antimicrobial activity of algae extracts towards *Salmonella* spp., *L. monocytogenes*, *E. coli*, *S. aureus* and *B. cereus* represented by heatmap. A scale ranging from a minimum of 0 mm (green) to a maximum of 15 mm (red) was used to represent size of inhibition diameter calculated, as average values of triplicates, after 24 h (**A**), 48 h (**B**), and 120 h (**C**) of incubation at 37 °C. I and II represent two different extracts referred to the same algae species.

The in vitro antimicrobial activity of the extracts was evaluated by agar well diffusion assay as described by Martelli et al. (2020) [19]. This test is based on the measurement of the inhibition zone on a bacterial cells layer, after the spreading in the culture medium of the extracts to be tested. Fourteen foodborne pathogenic strains belonging to *Salmonella* spp., *L. monocytogenes*, *E. coli*, *S. aureus*, and *B. cereus* were considered. The extracts’ activities were determined by evaluating the dimension of the inhibition zone (mm in diameter) around the well after 24, 48, and 120 h of incubation at 37 °C. In Figure 1 the heatmap representing in color scale the inhibition effect towards each foodborne pathogen along time is reported.

Overall, extracts obtained from *A. platensis* were the most effective. By comparing the antimicrobial activity after 24 and 48 h (Figure 1A,B) an overall loss of effectiveness was observed during incubation time of almost all the extracts. After 120 h (Figure 1C) some of them, in particular those deriving from *U. pinnatifida,* were no longer active toward the considered food pathogens.

In the case of *H. elongata, Laminaria* spp., *P. palmata* and *U. pinnatifida*, according to the statistical model, the antibacterial activity varied among extracts I and II obtained by the same seaweed species according to incubation time and to the pathogenic species (*p* value < 0.05). Only in the case of *A. platensis* did the extract antibacterial activity not vary significantly (*p* value ˃ 0.05).

**Figure 2:**
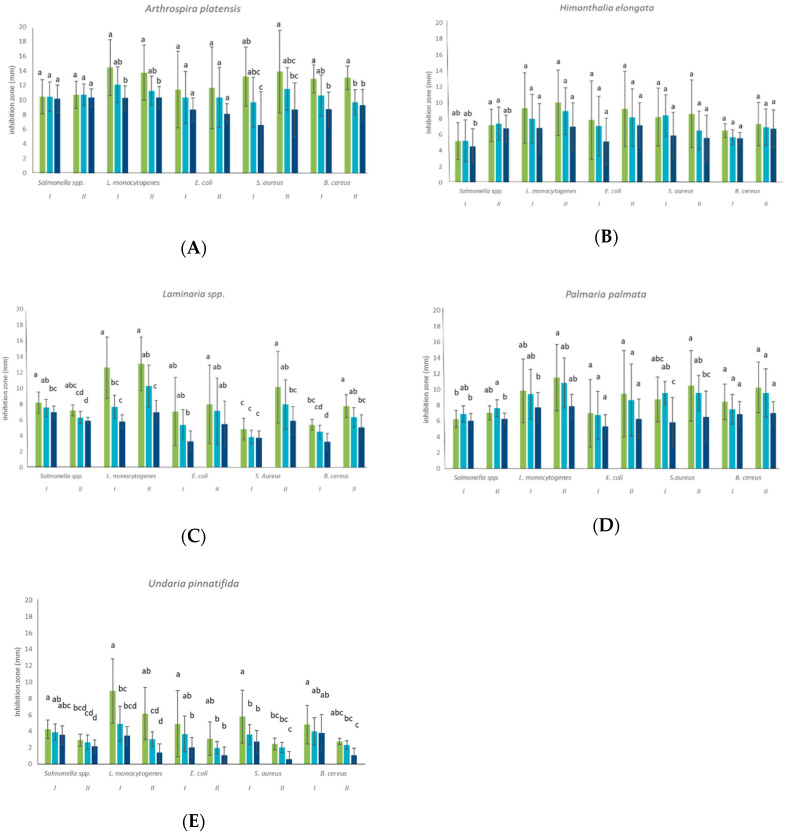
Antimicrobial activity of algae extracts along time: 24 h (Green line), 48 h (light blue line) and 120 h (Blue line). (**A**) *Arthrospira platensis* extracts; (**B**) *Himanthalia elongata* extracts. (**C**) *Laminaria* spp. extracts. (**D**) *Palmaria palmata* extracts. (**E**) *Undaria pinnatifida* extracts. The means with different letters are significantly different (*p* < 0.05).

In Figure 2 the activity of each extract against the tested foodborne pathogens is reported.

The high standard deviation values underlined the heterogeneous efficacy of the extracts inside the same population. The inhibition of *Salmonella* spp. by both *A. platensis* extracts did not significantly differ, and the effect remained constant during time (Figure 2A). The same behaviour was observed regarding *E. coli*. In the case of *L. monocytogenes*, both extracts led to a significant decrease in the inhibition zone along time, resulting in a loss of effectiveness (*p* < 0.05). The behaviour of *S. aureus* and *B. cereus* was very similar to *L. monocytogenes* when *A. platensis* extracts were used. However, extracts I and II exerted inhibitory effects differently on *S. aureus* and, in particular, a significant difference in the first 24 h occurred (*p* < 0.05).

The inhibitory zone of *H. elongata* extracts towards *Salmonella* spp. remained constant after 24 and 48 h of incubation, then decreased after 120 h, highlighting a loss of effectiveness in the last three days of observation (Figure 2B). In addition, a significant difference between the activity of extracts was highlighted (*p* < 0.05). The antibacterial activity of *H. elongata* extracts against all the other food pathogens (*L. monocytogenes, E. coli, S. aureus and B. cereus*) did not show any significant differences either as a function of incubation time or according to the origin of the sample.

The antimicrobial activity of *Laminaria* spp. extracts was particularly high towards *L. monocytogenes*, but it decreased significantly over time (*p* < 0.05) (Figure 2C). When referring to *Salmonella* spp. and *B. cereus,* a significant difference between the effectiveness of the two extracts (*p* < 0.05) could be noticed. The inhibition of *L. monocytogenes* was different when considering extracts I and II at 48 h. In Figure 2C it can also be observed that the inhibition of *E. coli* remained constant in extract I for the first 48 h, and then decreased. The two extracts showed a significantly different activity towards *S. aureus* (*p* < 0.05).

The inhibition of *Salmonella* spp. due to *P. palmata* extracts I and II was constant in the first 48 h (Figure 2D). The *L. monocytogenes* case was different: the inhibitory zone remained constant in the first 48 h for extract I and then decreased, while in the presence of extract II after a halo first decrease at 24 h, it remained stable. The inhibition of *P. palmata* extracts against *E. coli* and *B. cereus* was constant along time with any significant difference. Extract I behaved towards *S. aureus* in a similar way to *Salmonella*.

*U. pinnatifida* extracts showed the lower antimicrobial activity when compared to all the tested extracts (Figure 2E).

### 3.2. Evaluation of Antimicrobial Activity In Situ by Microbiological Challenge Test

In order to evaluate the antimicrobial effect of algal extract in situ, a contamination simulation with a mixture of *L. monocytogenes* strains was performed on salmon tartare chosen as food model. For this purpose, *A. platensis* extract II, which showed the highest antimicrobial activity in vitro, was selected as a useful ingredient for the improvement of microbiological food safety. Two concentrations of extract were considered in the formulation: 0.45 and 0.9% (*v*/*w*).

**Figure 3:**
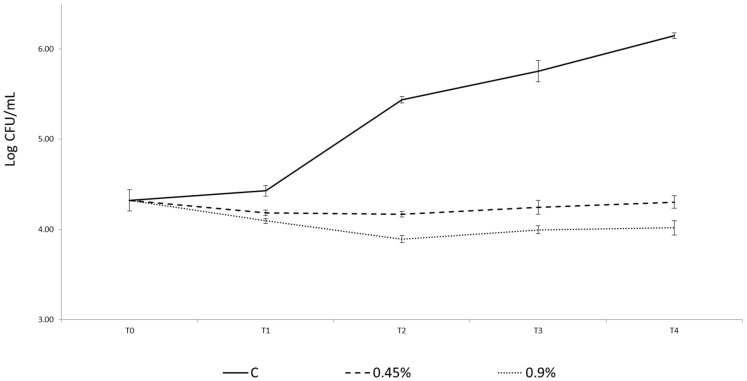
Behavior of *Listeria monocytogenes* during four days’ shelf life at refrigeration temperatures in salmon tartare without extract addition (C) (―), with 0.45% (*v*/*w*) (---) and 0.9% (*v*/*w*) (∙∙∙∙∙) of *A. platensis* extract.

In the control sample (C), without extract addition, *L. monocytogenes*, artificially inoculated in order to reach an initial contamination of 4 Log cfu/g in the product, remained at the inoculum level during the first 24 h and then rapidly grew by two logarithms (Figure 3). So a completely different behavior of the pathogen in the presence of 0.45% and 0.9% (*v*/*w*) of extract was evident (Figure 3). Indeed, in these samples the contamination was maintained at the inoculum level during four days of storage, proving a bacteriostatic activity of the extract.

### 3.3. Phenolic Content

The amount of total phenolic compounds of the different algae extracts is reported in Table 1. Among the samples tested, *U. pinnatifida* presented the lowest phenolic content, while the highest was found in *H. elongata* extracts. Regarding these latter, a significant higher phenolic content was found in extract II in respect to all the other considered samples (18.79 ± 1.90 mg GAE/g). Extracts obtained from the red algae specie *P. palmata* and the cyanobacterium *A. platensis* showed comparable phenolic content, and presented similar results as the second group in terms of phenol abundance, with values of 2.45 ± 0.06–4.56 ± 0.41 mg GAE/g and 3.18 ± 0.48–3.40 ± 0.42 mg GAE/g respectively. Significant lower quantities of phenols were found in *Laminaria* spp. and in *U. pinnatifida* extracts, both pertaining to the group of brown algae. In particular, *U. pinnatifida* contained 10 times less phenolic compounds in respect to all the other seaweed species considered, with values of 0.15 ± 0.01 mg GAE/g (Table 1).

## 4. Discussion

Thanks to good nutritional value and high bioactive compound content, the use of seaweeds in food and feed formulation is growing everyday [26,27]. Seaweeds and derived compounds have proved a series of bioactivities that could significantly enhance the use of these organisms in food formulation in order to guarantee a healthy diet for consumers [28]. In particular, antioxidant, anti-inflammatory, anti-cancer, and anti-diabetic properties are attributed to compounds extracted from seaweeds and microalgae. Favoring the requests of corporations and consumers for clean label products, researchers are more interested nowadays in the compounds extracted from plants and, among these, to new antimicrobial agents [29]. In this context, currently seaweeds and algae may represent a promising resource, even if many of the extracts obtained up to now are derived from species not recognized as edible. The possibility of obtaining novel antimicrobial compounds from seaweeds and microalgae, already approved by EFSA as food, enhance their applications in food formulations. Furthermore, these extracts could not only work as antimicrobial compounds, but also as a source of flavors. Different works have focused on the in vitro antimicrobial activity of several algae extracts against *Salmonella* spp. [30,31,32,33,34,35,36], *L. monocytogenes* [19,25,37,38], *E. coli* [39,40,41,42], *S. aureus* [43,44,45,46] and *B. cereus* [47], demonstrating different effectiveness depending on the algae species and extraction process. Others have reported antimicrobial activity referring to the algae species tested in the present study, even if a different extraction process was followed. The extraction protocol used in this study showed a good antimicrobial activity, contrary to other studies which state that the use of a methanolic solution or dichloromethane is better to extract antimicrobial compounds from seaweeds [48]. Moreover, to the best of our knowledge, the efficacy of the extracts in situ using a microbiological challenge test has not yet been tested. Numerous macro and microalgae have been discussed for their content in bioactive compounds [16,30] among which polysaccharides, proteins and amino acids, polyunsaturated fatty acids (PUFAs) and antioxidants (polyphenols, carotenoids and flavonoids) demonstrated antimicrobial activity [49,50,51]. This last group of secondary metabolites has been associated with a broad spectrum of in vitro antibacterial activity, specifically against *Bacillus* spp. and *S. aureus*. Phlorotannins are the most important phenolic compounds characteristic of brown seaweeds. Other phenolic compounds, such as bromophenols, flavonoids, phenolic acids, anthraquinones, coumarins, rutin, quercetin, and kaempferol, present in different algae species, have also demonstrated antimicrobial potential [16,49,52]. The antimicrobial activity of algae against both Gram-positive and Gram-negative bacteria was also traced back to fatty acids [53] that represent a defense against bacteria, viruses and protozoans [54].

Each type of algae, because of various classes and quantities of compounds, has a different inhibitory activity on diverse classes of bacteria [55]. In this study, all the algae species considered (*A. platensis*, *H. elongata*, *Laminaria* spp., *P. palmata* and *U. pinnatifida*) have generally shown a good antimicrobial activity (Figure 2). However, differences in the efficacy of the extracts against Gram positive and Gram negative bacteria have arisen. The cyanobacterium extracts showed the highest antimicrobial activity against Gram positive bacteria (*L. monocytogenes*, *S. aureus* and *B.cereus*) as already demonstrated [56], but an interesting effect was also observed on Gram negative bacteria. *H. elongata* extracts were highly effective against the Gram positive bacteria *L. monocytogenes* but strong differences between Gram positive and Gram negative were not noticed. Similarly another *Phaeopyceae, U. pinnatifida*, proved the highest efficacy against *L. monocytogenes. Laminaria* spp. extracts mainly inhibited two Gram positive bacteria, *L. monocytogenes and S. aureus.* A higher antimicrobial activity in Gram positive as opposed to Gram negative was also observed in the *Rodophyta P. palmata* extracts. These results confirm those from other studies, in which the highest antimicrobial activity of seaweed extracts is found against Gram positive bacteria [43,57]. *A. platensis* has been studied as a valuable source of antimicrobial, antiviral and antioxidant compounds but its activity is variable and dependent on the extractive solvent [58,59,60]. This cyanobacterium, commercially known as Spirulina, is used in food formulations or as a food supplement [6,61] and in health products [62,63,64,65], because of its composition rich in proteins and small peptides. The antimicrobial potential of small peptides is well known and the activity of several algae is related to their high protein content. The high antimicrobial activity of *A.platensis* extracts I and II could definitively be linked to the large amount of proteins and small peptides of this cyanobacterium (Figure 2A). The application of an *A. platensis* extract as preservative, as far as the authors are aware, has never been tested in situ and could find applications, for instance, in seafood products due to its sea like smell (Figure 3). In particular, these products are frequently subjected to *L. monocytogenes* contaminations and their psycho-trophic behavior. In the first six months of 2020, RASFF (The Rapid Alert System for Food and Feed) has issued seven warnings regarding *L. monocytogenes* contaminating salmon based products (RASFF 2020.0525, 2020.0683, 2020.0876, 2020.1049, 2020.1631, 2020.2276 and 2020.2409). The behavior and the growth ability of this microorganism in salmon based food is widely studied [66]. Salmon is generally preserved under pressure packaging or CO_2_ atmosphere packaging, but the use of natural origin preservatives is a well-considered possibility [67]. Recently, the efficacy of essential oils (EOs) obtained from plants and fruits has been evaluated with interesting results in terms of MIC (Minimal Inhibitory Concentration) and MBC (Minimal Bactericidal Concentration) referring to *L. monocytogenes* strains isolated from salmon [68], though, from a sensory point of view, the use of EOs was not very compatible with salmon as they masked fish or seafood odor and flavor. For that reason, the use of an alternative with a sea like flavor could be applicable, apart from being effective. Indeed, an *A. platensis* extract obtained by using the extraction protocol of the present study was tested against *Listeria innocua* and a MIC of 0.20% and a MBC of 0.30% were found, proving good efficacy [69]. *A. platensis* extracts have already been tested against several food pathogenic bacteria, though those studied ed in this work proved a higher antimicrobial activity against *B. cereus* compared to other studies [41]. In fact, both ethanolic and aqueous extract didn’t exert any inhibition on the pathogenic bacteria.

*Phaeophyceae* is described in literature as the class of seaweeds with the most relevant antimicrobial activity. Promising groups of polysaccharides, such as Fucoidans and other sulphated polysaccharides extracted from *Phaeophyta*, have demonstrated an antimicrobial potential. In particular, sulphated polysaccharides and, among them, alginates, fucoidans and laminarian, inhibited the growth of *E. coli* and *S. aureus* [70]. Fucoidans have shown not only antimicrobial activity against food pathogenic bacteria but also antiviral, antioxidant, immunomodulatory, anticoagulant, antithrombotic, antitumor and anti-inflammatory activities [71,72]. In this study three species belonging to *Phaeophyceae* have been tested. The antimicrobial activity of *H. elongata* extract has been previously studied with similar results [19,73,74], while the extracts obtained in the present work have been more effective against *L. monocytogenes* and *Salmonella* spp. than those obtained by Rajauaria et al. [74] (Figure 2B). In fact, the extract produced an inhibition halo of 9.95 mm, while the extract obtained in the present work led to a halo of more than 10 mm. The genus *Laminaria*, characterized by *Phaeophyceae* of large dimensions, also proved a good antimicrobial potential towards *L. monocytogenes, S. aureus* and *Salmonella ebony* [25,44], even if a different extraction protocol was used. In fact, the methanolic *Laminaria* spp. and *H. elongata* extracts obtained by Cox and colleagues (2010) ], have shown a very valuable antimicrobial activity against the food pathogenic bacteria tested [25]. These algal extracts were able to inhibit the growth of *L. monocytogenes* by 100%. Indeed, the food grade extraction method applied in the present work was never tested on this species and led to interesting results (Figure 2C). Otherwise, *U. pinnatifida* did not reveal a satisfactory antimicrobial activity and this could be due to the extraction process (Figure 2E). Its antimicrobial activity has been connected to essential oils content [75], but in this case a polar solvent, water and ethanol, was in fact applied.

*P. palmata* is a red seaweed characteristic of the Atlantic ocean and it also demonstrated a good antimicrobial activity against *L. monocytogenes*, *Salmonella ebony, Enterococcus faecalis* and *Pseudomonas aeruginosa* [25]. No information about the antimicrobial effect of *P. palmata* extracts on *E. coli, B. cereus* or *S. aureus* are reported in the literature (Figure 2D). It can be supposed that the extraction procedure applied in this study obtained extracts rich in polyphenolic compounds. The tested algae and the extraction process can also indicate that the antimicrobial activity could be due to small peptides, polysaccharides and phenolic compounds that act synergistically. Indeed, phenolic content appears not to be the main component of the activity we observed, though some relations could be noticed. *H. elongata* extract II, having the highest phenolic content (18.79 ± 1.90 mg GAE/g), had a significantly higher antimicrobial activity (*p* < 0.05) if compared to *H. elongata* extract I (1.52 ± 0.38 mg GAE/g) towards *Salmonella* spp., presenting a difference in inhibition halo higher than 2 mm (Figure 2B). *A. platensis* extracts did not prove a significant difference (*p* > 0.05) on antimicrobial activity (Figure 2A), nor in phenolic content (Table 1). Regarding *Laminaria* extracts, the differences in antimicrobial activity cannot be related to the phenolic content, which did not differ (Figure 2C). Otherwise, the different effects of *P. palmata’s* extracts towards *L. monocytogenes, Salmonella* spp. and *S. aureus* (*p* < 0.05) (Figure 2D) might be linked to their phenolic content which significantly differ (*p* < 0.05) (4.56 ± 0.41 and 2.45 ± 0.06 mg GAE/g respectively for extract I and II) (Table 1). Finally, the near absence of phenolic compounds in *U. pinnatifida* extracts corresponded to a low antimicrobial activity of these samples (Figure 2E). The protocol used to obtain the extracts does not involve the use of high temperature, and for that reason the phenolic content of the matrix should not be affected during the extraction process [76].

The results obtained showed that the phenolic content of seaweed seems to be related to the species, as also reported in previous studies. Cox et al. (2010) analysed the phenolic content in seaweed samples belonging to different species, obtaining values ranging from about 37 mg GAE/g for *L. digitata* to 151 mg GAE/g for *H. elongata*. In that case, the authors speculated that total phenolic content could vary among brown, red or green seaweeds. Brown seaweeds have been described as rich in polyphenols [77] and *H. elongata* was found as the species with the highest total phenolic amount [52], as reported also in the present study. In addition, it has to be taken into account that the concentration of phenols may present a wide range of variability in these kind of matrices. It was indeed demonstrated that total phenolic amount was strongly influenced by several factors such as seasonality, temperature, light intensity, and water salinity [78]. This may explain why *H. elongata* extracts I and II showed a significant different phenolic content (Table 1). Even if to a less extent, a similar trend was observed in the case of the red seaweed *P. palmata*: extract I presented a significant higher phenolic content (4.56 ± 0.41 mg GAE/g) compared to extract II in which this concentration resulted in about a half of that (2.45 ± 0.06 mg GAE/g) (*p* < 0.05). Taking onto account that samples were provided by two different suppliers, it can be supposed that the considered seaweed pertaining to *H. elongata* and *P. palmata* have been subjected to diverse stabilization treatments before commercialization or have been collected from different areas and/or in different seasons. Concerning *A. platensis*, *Laminaria* spp. and *U. pinnatifida* no differences were found between total phenolic amount of the two extracts inside the species, but the content resulted in significant differences among the species. In particular, phenolic content of *A. platensis* extracts of 3.29 ± 0.45 mg GAE/g was comparable with data recently obtained by da Silva et al. (2017) [79] who reported a total amount of 3.32 ± 0.08 mg GAE/g in samples of *A. platensis* extracted with high pressure/temperature extraction (HPTE) method. Total phenolic content of *Laminaria* spp. calculated in the present work (I:1.15 ± 0.25 and II:1.55 ± 0.92 mg GAE/g) (Table 1) showed results in line with data reported by Rodríguez-Bernaldo de Quirós et al. (2010) [52], that showed an amount of 1.3 ± 0.6–1.4 ± 1.0 expressed as mg Phloroglucinol/g. Conversely, the same authors reported a value of 6.0 ± 2.2 mg Phloroglucinol/g for *U. pinnatifida*, while lower amounts were found in this study for the same variety (0.15 ± 0.01 mg GAE/g).

## 5. Conclusions

In order to ensure microbiological food safety and improve quality according also to consumer preferences for “clean label” products, food corporations are looking for new compounds to be used as an alternative solution to traditional preservatives. This study highlighted that: (i) extracts obtained from different species of edible algae exerted antimicrobial activity towards the main foodborne pathogens such as *Salmonella* spp., *L. monocytogenes*, *E. coli*, *S. aureus*, and *B. cereus*; (ii) *A. platensis* extract showed an interesting antibacterial activity in situ towards *L. monocytogenes*; (iii) a great variability in the phenolic content of the tested seaweed was noticed, and this content was not always related to the antimicrobial activity observed, emphasizing the possibility of a synergic effect involving other compounds.

In this context, a deeper investigation of extract composition in order to evaluate the effect of compounds responsible for the antimicrobial activity will surely be of great interest. Overall, the effectiveness of algae extracts was confirmed even if results suggested that antibacterial activity can be influenced by various factors: it varies according to algal species, producers, contact time and foodborne pathogens. In particular, the growth inhibitory efficacy toward *L. monocytogenes* of *A. platensis* extract, highlighted by the microbiological challenge test, could open up perspectives on applications for food preservation. Indeed, this antimicrobial extract might work as food preservative and antioxidant not only in fish products but also in other products that need to guarantee safety criteria and to preserve freshness by slowing down microbial growth.

## Figures and Tables

**Table 1:** Total phenolic content (TPC) of seaweed extracts expressed as mg GAE/g.

Algae Species	TPC (mg GAE/g)
*Himanthalia elongata* I	1.52 ^d^ ± 0.38
*Himanthalia elongata* II	18.79 ^a^ ± 1.90 *
*Arthrospira platensis* I	3.18 ^bc^ ± 0.48
*Arthrospira platensis* II	3.40 ^bc^ ± 0.42
*Laminaria* spp. I	1.15 ^d^ ± 0.25
*Laminaria* spp. II	1.55 ^d^ ± 0.92
*Palmaria palmata* I	4.56 ^b^ ± 0.41 *
*Palmaria palmata* II	2.45 ^c^ ± 0.06
*Undaria pinnatifida* I	0.16 ^e^ ± 0.01
*Undaria pinnatifida* II	0.14 ^e^ ± 0.01

* indicate a significant difference (*p* < 0.05) between samples pertaining to the same specie (*T*-test), while letters highlight differences and/or analogies among different species (one way Anova).
